# Bronchopleural Fistula Following Lobectomy after Prior Thoracic Endovascular Aortic Repair: A Case Report

**DOI:** 10.70352/scrj.cr.25-0458

**Published:** 2025-09-27

**Authors:** Kohei Abe, Jun Suzuki, Hikaru Watanabe, Satoshi Takamori, Takayuki Sasage, Tetsuro Uchida, Satoshi Shiono

**Affiliations:** Department of Surgery 2, Faculty of Medicine, Yamagata University, Yamagata, Yamagata, Japan

**Keywords:** complication, thoracic endovascular aortic repair, bronchopleural fistula, lobectomy

## Abstract

**INTRODUCTION:**

Bronchopleural fistula (BPF) is a severe complication of lung cancer surgery that is often associated with impaired blood supply and poor healing. Thoracic aortic aneurysms (TAA) frequently coexist with lung cancer owing to their shared risk factors, and thoracic endovascular aortic repair (TEVAR) may further increase the risk of BPF by compromising bronchial artery blood flow.

**CASE PRESENTATION:**

A 72-year-old male with a history of cerebral infarction, hypertension, and a 4.8 cm TAA was incidentally found to have a 1.4 cm pulmonary nodule in the right lower lobe. Staged treatment was planned using TEVAR, followed by right lower lobectomy. TEVAR was performed successfully, and 48 days later, lobectomy confirmed stage IB large-cell carcinoma. On POD 27, the patient developed fever and dyspnea. CT revealed BPF, which was likely due to reduced bronchial blood flow after TEVAR. Chest tube drainage and an open-window thoracostomy were performed. Eight months later, thoracoplasty was performed using a muscle flap and omentopexy. The patient recovered completely and remained recurrence-free for 2 years.

**CONCLUSIONS:**

This case underscores the importance of careful planning and surgical strategies to prevent BPF in patients with concomitant lung cancer and TAA. Early recognition of risk factors, including TEVAR-related bronchial ischemia, is crucial for optimizing outcomes.

## Abbreviations

BPF
: bronchopleural fistula
TAA
: thoracic aortic aneurysms
TEVAR
: thoracic endovascular aortic repair

## INTRODUCTION

A bronchopleural fistula (BPF) is an intractable complication of lung cancer surgery. Impaired blood supply or poor wound healing mechanisms could be regarded as potential risk factors for the development of BPF. Since the risk factors for thoracic aortic aneurysm (TAA) may partially overlap with those for lung cancer, the coexistence of TAA and lung cancer can occur. Specific and meticulous management is mandatory for cases of lung cancer concomitant with TAA. Herein, we report the case of a patient with a history of thoracic endovascular aortic repair (TEVAR) who developed postlobectomy BPF 1 month after TEVAR.

## CASE PRESENTATION

**Fig. 1:**
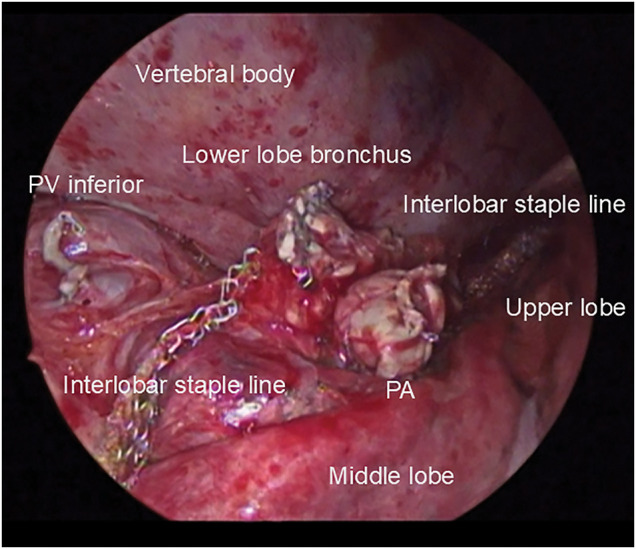
Intraoperative photograph of the hilum after lower lobectomy. The cut ends of the inferior PV, PA, and lower lobe bronchus are visible. The interlobar staple line is also clearly seen. PA, pulmonary artery; PV, pulmonary vein

**Fig. 2:**
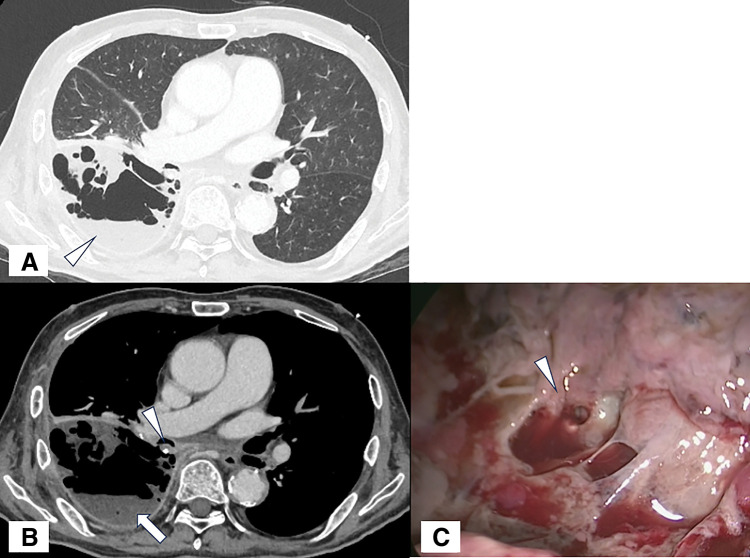
Findings of a bronchial stump fistula. (**A**) On lung window CT, lung collapse and pleural effusion (arrowhead) were observed. (**B**) On mediastinal window CT, pleural effusion and air accumulation in the right thoracic cavity (arrow) were observed, along with findings suggestive of communication between the bronchial and pleural cavities (arrowhead). (**C**) An approximately 1.5 cm fistula in the bronchial stump (arrowhead).

The patient was a 72-year-old male with a history of cerebral infarction and hypertension. He smoked one pack of cigarettes per day for 48 years until he quit smoking 4 years ago. He had no history of diabetes mellitus, nor did he use steroids or immunosuppressive drugs. Laboratory tests showed a hemoglobin level of 13.5 g/dL and an albumin level of 3.7 g/dL. During the assessment of bloody stool, TAA (saccular type, 4.8 cm in diameter) and a pulmonary nodule were incidentally identified on CT. The pulmonary nodule was 1.4 cm in size and was located in the right lower lobe. PET/CT revealed a maximum standardized uptake value of 2.3. Radiological findings suggested that the pulmonary nodule was lung cancer, although the biopsy of the nodules was restrained due to TAA. After a multidisciplinary consultation, we decided to perform staged surgery for the TAA and lung nodules. TEVAR was planned for the TAA, followed by surgery for the pulmonary nodule. TEVAR was successfully performed with a smooth recovery, and the patient was referred to our department 19 days later. Lung surgery was performed 48 days after TEVAR, and a frozen section diagnosis of the nodule confirmed lung adenocarcinoma. Consequently, a thoracoscopic right lower lobectomy with mediastinal lymph node dissection was performed. The lymph node dissection included both hilar nodes and mediastinal stations 7 through 9. No injury to the bronchial arteries was observed. The right lower bronchus was transected using a stapler (**Fig. 1**). The bronchial stump was then covered with a polyglycolic acid sheet and a fibrin sealant. The surgery time was 225 min, with a blood loss of 140g. Intraoperatively, there was no evidence of tissue fragility or increased bleeding tendency. No abnormal course of the bronchial arteries was observed intraoperatively. Only the bronchial arteries required for resection of the lower lobe bronchus were divided. The postoperative course was uneventful, and the patient was discharged on POD 4. The pathological diagnosis confirmed a large cell carcinoma (pT2aN0M0-Stage IB). On POD 27 after the lung cancer surgery, the patient complained of fever and dyspnea and visited the emergency department. His initial vital signs were as follows: body temperature: 37.8°C, blood pressure: 127/52 mmHg, heart rate: 101/min, respiratory rate 46/min, and SpO2: 91% in room air. Laboratory tests revealed an elevated white blood cell count of 16770/μL and a C-reactive protein level of 12.48 mg/dL. Chest radiography revealed a decreased translucency of the right lower lung field. CT showed increased fluid level and air space in the right thoracic cavity, suggesting BPF (**Fig. 2A**, **2B**). Chest tube drainage was performed and the patient received antimicrobial therapy. Since a massive air leak was observed in the chest tube, an open-window thoracostomy was performed 2 days after readmission. Intraoperative findings revealed pleural adhesions in the remaining lung, bloody pleural effusion, and an approximately 1.5 cm fistula in the bronchial stump (**Fig. 2C**). After open-window thoracostomy, the patient recovered smoothly and was discharged on POD 34 after open-window thoracostomy. Thoracoplasty was performed 8 months after the open window procedure. An erector spinae muscle flap was sutured to the fistula and omentopexy was performed. The patient was discharged on POD 7 after thoracoplasty and has remained recurrence-free for 2 years.

**Fig. 3:**
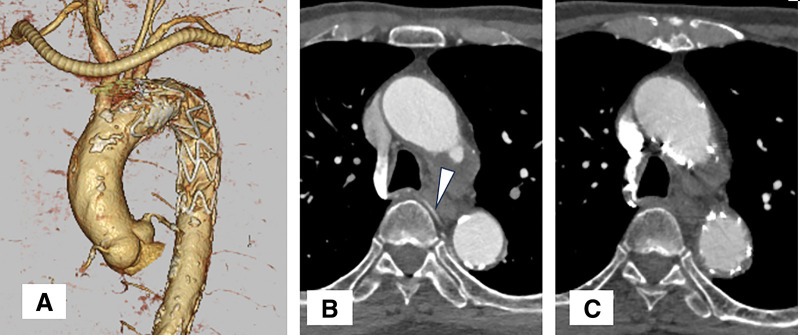
(**A**) The origin of the intercostal artery was broadly covered by the stent graft. (**B**) On the CT before TEVAR, the right 4th intercostal artery (arrowhead) was clearly identified. (**C**) On the CT after TEVAR, the right 4th intercostal artery could not be identified. TEVAR, thoracic endovascular aortic repair

The cause of BPF was discussed during the clinical course of treatment. 3D images showed that the origin of the intercostal artery was covered by the stent graft after TEVAR, and a decrease in bronchial artery blood flow was anticipated (**Fig. 3A**). We checked contrast CT before and after TEVAR. The bronchial artery started from the right 4th intercostal artery and was seen before TEVAR (**Fig. 3B**). However, it was not seen on the CT before lobectomy after TEVAR (**Fig. 3C**).

## DISCUSSION

BPF is one of the most serious complications encountered by thoracic surgeons, occurring in approximately 0.44%–2.4% of patients undergoing lung tumor resection.^1–3)^ According to an annual Japanese report, BPF is the 6th cause of death after lung cancer.^4)^ A recent review indicated that the incidence of BPF is associated with the type of surgical procedure, including subcarinal lymph node dissection, preoperative therapy such as chemotherapy and radiotherapy, postoperative complications, right side, elderly patients, history of gastric cancer,^1,2)^ hypoalbuminemia, and anemia.^5)^ Diabetes mellitus is also a risk factor for BPF.^6)^

Bronchial mucosal ischemia is thought to play an important role in BPF.^7)^ A meta-analysis of neoadjuvant therapy for lung cancer revealed a relationship between neoadjuvant therapy and BPF. This suggests that insufficient blood flow after neoadjuvant radiotherapy and chemoradiotherapy can affect the development of BPF.^3)^ After TEVAR, the origin of the bronchial artery may be covered by the stent graft, potentially reducing the blood flow to the bronchial stump and contributing to the development of BPF. Occlusion of the bronchial artery can affect lung function and circulation. Hino et al.^8)^ noted that bronchial arteries from the descending thoracic aorta are vital for bronchial stump perfusion, and disruption by TEVAR or mediastinal lymph node dissection may impair healing and predispose to BPF. One study suggested the possibility of pulmonary edema due to bronchial artery occlusion.^9)^ Hence, we hypothesized that interruption in the blood flow by TEVAR could lead to bronchial ischemia and BPF. Only one previous report has documented the occurrence of BPF after pulmonary resection following TEVAR.^8)^ In this report, we suggest the possibility of bronchial artery occlusion due to TEVAR. This is the second reported case of BPF following TEVAR. Both patients were men in their 70s who had undergone right lower lobectomy with lymph node dissection. These findings suggest that prior TEVAR may increase the risk of BPF, in addition to previously documented factors. Since the number of such cases is currently too low to provide a definitive explanation, TEVAR is considered a possible risk factor for BPF. However, as the bronchial artery is frequently sacrificed during lymph node dissection, we should not only focus on the effect of TEVAR but also consider other factors related to BPF. In this case, in addition to the history of TEVAR, male sex, right lower lobectomy, and subcarinal lymph node dissection were considered risk factors for BPF. However, there was no history of diabetes mellitus or steroid use, nor were any intraoperative bronchial artery injuries or abnormalities observed.

Since partially overlapping risk factors may exist between TAA and lung cancer, the coexistence of these 2 diseases is not uncommon. Pasqui et al.^10)^ reported that among 148 lung cancer patients, TAA was observed in 17 (12%). In cases of lung cancer concomitant with TAA, despite TAA treatment and perioperative management, careful preoperative planning for BPF is required. Habu et al.^11)^ reported the significance of bronchial coverage using a thick flap for reducing the risk of BPF. Based on our experience with this case, although scientific evidence remains limited, bronchial coverage using a thick flap should be considered in patients with lung cancer concomitant with TAA. Additionally, when feasible, it may be advisable to delay lung cancer surgery until after TEVAR or to perform lung cancer surgery prior to TEVAR.

## CONCLUSIONS

This case demonstrates the need for caution in managing patients with concomitant lung cancer and TAA, particularly regarding the risk of BPF following TEVAR and lobectomy. Therefore, careful consideration is required when planning lung resection after TAA treatment.

## DECLARATIONS

### Availability of data and materials

The case report and patient consent form are available from the corresponding author upon reasonable request.
